# Fall Prevention for Rural Spanish-Speaking Communities: Pilot Study

**DOI:** 10.1093/geroni/igaf122.4351

**Published:** 2025-12-31

**Authors:** Janet Lopez, Ladda Thiamwong, Rui Xie, Victoria Loerzel

**Affiliations:** University of Central Florida, Orlando, Florida, United States; University of Central Florida, Orlando, Florida, United States; University of Central Florida, Orlando, Florida, United States; University of Central Florida, Orlando, Florida, United States

## Abstract

This community-engaged research focuses on the information gathering phase for culturally adapting the Physio-feedback Exercise Program (PEER) for middle-aged and older Spanish-speaking adults in rural Central Florida communities. PEER is a technology-based intervention combining real-time balance feedback using the BTrackS Balance System, cognitive reframing through a fall risk appraisal matrix, and peer-led balance and strength training exercises. While PEER has demonstrated effectiveness in urban community-dwelling older populations, rural communities face distinct barriers including limited technology access, different cultural contexts, and unique health challenges related to falls and frailty. Using the Heuristic Framework and Ecological Validity Model, this study presents the first phase of a four-step cultural adaptation process: information gathering. Through participatory methods involving community partners, we collected comprehensive data about rural Spanish-speaking middle-aged and older adults (N = 30) to inform subsequent PEER adaptation. We evaluated community needs for fall prevention and conducted a rapid analysis. Findings revealed that most participants were female (n = 22) and had a lower than high school education (n = 24). Some indicated that they had never heard of fall prevention programs or did not understand their purpose. None were aware of such programs in their communities. Many expressed interest in participating in fall prevention programming. They emphasized the importance of providing these programs in Spanish, ensuring scheduling flexibility (including evenings and weekends), and offering free programming. This gathering of information focuses on adapting evidence-based interventions for underserved rural populations. These findings will directly inform the cultural adaptation of PEER for middle-aged and older Hispanic adults in rural communities.

